# Generation of Corneal Epithelial Cells from Induced Pluripotent Stem Cells Derived from Human Dermal Fibroblast and Corneal Limbal Epithelium

**DOI:** 10.1371/journal.pone.0045435

**Published:** 2012-09-24

**Authors:** Ryuhei Hayashi, Yuki Ishikawa, Miyuki Ito, Tomofumi Kageyama, Kuniko Takashiba, Tsuyoshi Fujioka, Motokazu Tsujikawa, Hiroyuki Miyoshi, Masayuki Yamato, Yukio Nakamura, Kohji Nishida

**Affiliations:** 1 Department of Ophthalmology, Osaka University Graduate School of Medicine, Suita, Osaka, Japan; 2 Department of Ophthalmology, Tohoku University Graduate School of Medicine, Sendai, Miyagi, Japan; 3 Cell Engineering Division, RIKEN BioResource Center, Tsukuba, Ibaraki, Japan; 4 Subteam for Cell Fate Manipulation, RIKEN BioResource Center, Tsukuba, Ibaraki, Japan; 5 Institute of Advanced Biomedical Engineering and Science, Tokyo Women's Medical University, Tokyo, Japan; Osaka University Graduate School of Medicine, Japan

## Abstract

Induced pluripotent stem (iPS) cells can be established from somatic cells. However, there is currently no established strategy to generate corneal epithelial cells from iPS cells. In this study, we investigated whether corneal epithelial cells could be differentiated from iPS cells. We tested 2 distinct sources: human adult dermal fibroblast (HDF)-derived iPS cells (253G1) and human adult corneal limbal epithelial cells (HLEC)-derived iPS cells (L1B41). We first established iPS cells from HLEC by introducing the Yamanaka 4 factors. Corneal epithelial cells were successfully induced from the iPS cells by the stromal cell-derived inducing activity (SDIA) differentiation method, as Pax6^+^/K12^+^ corneal epithelial colonies were observed after prolonged differentiation culture (12 weeks or later) in both the L1B41 and 253G1 iPS cells following retinal pigment epithelial and lens cell induction. Interestingly, the corneal epithelial differentiation efficiency was higher in L1B41 than in 253G1. DNA methylation analysis revealed that a small proportion of differentially methylated regions still existed between L1B41 and 253G1 iPS cells even though no significant difference in methylation status was detected in the specific corneal epithelium-related genes such as K12, K3, and Pax6. The present study is the first to demonstrate a strategy for corneal epithelial cell differentiation from human iPS cells, and further suggests that the epigenomic status is associated with the propensity of iPS cells to differentiate into corneal epithelial cells.

## Introduction

Like embryonic stem cells, induced pluripotent stem (iPS) cells are capable of differentiating into all of the various cell lineages of an organism, and are established from somatic cells by introducing transcription factors such as Oct3/4, Sox2, and Klf4 [1]–[3]. Therefore, iPS cells can be used as a cell source to regenerate tissues, such as retinal pigment epithelium (RPE), neurons, cardio muscle tissue, and corneal epithelium, and have great potential resolve current issues in the transplant field such as donor shortages, immune rejection, and ethical controversy.

The cornea is transparent tissue located in the anterior chamber of eye that is composed of 3 layers: corneal epithelium, stroma, and endothelium. The corneal epithelium originates from surface ectoderm during development, similar to the epidermis or lens epithelium [4]. Their stem/progenitor cells are believed to localize in the basal epithelium of the limbus located between the cornea and conjunctiva [5], [6]. If corneal epithelial stem cells are completely absent due to limbal stem cell deficiencies, the peripheral conjunctival epithelium invades inwardly and the corneal surface is enveloped by vascularized conjunctival scar tissue, resulting in corneal opacification and blindness from this severe eye disease [7], [8]. Although transplant therapy has been performed in patients with corneal epithelial stem cell deficiencies, most failed due to immune rejection [9]. Regenerative medicine using differentiated autologous iPS cells has been proposed as a promising alternative; however, no differentiation strategy has been determined thus far.

Recently, human iPS cells have been established from various cell sources, including human dermal fibroblasts (HDF), keratinocytes, neural precursor cells, blood, pancreas, and testis [10]–[14]. However, these reports suggested that some of these iPS cells were limited in their differentiation capability, as they retained their original epigenetic characteristics and have the propensity to differentiate into the cell lineage originally used as cell source [15]–[17]. This also suggests that the generally used HDF-derived iPS cells may have limited capability to fully differentiate into other cell lineages, such as corneal epithelial cells. Human limbal epithelial cells (HLEC), in contrast, contain corneal epithelial stem/progenitor cells that may more easily differentiate into corneal epithelial cells. Thus, in this study, we attempted to establish iPS cells derived from HLECs and examine the ability of both HLEC- and HDF-derived iPS cells to differentiate into corneal epithelial cells.

## Materials and Methods

### Establishment of iPS cells from human corneal limbal epithelial cells (HLEC)

HLECs were harvested from 2 adult human corneoscleral rims (Northwest Lions Eye Bank, Seattle, WA), according to the previously described method [18]. Isolated limbal epithelial cells containing corneal epithelial stem/progenitor cells were cultured in NIH/3T3 conditioned medium. Lentivirus vectors loaded with the Yamanaka 4 factors Oct3/4, Sox2, c-Myc, and Klf4 were used to reprogram limbal epithelial cells [19] (Table 1). All experiments using recombinant DNA were approved by the Recombinant DNA Committees of Osaka and Tohoku University and performed according to the institutional guidelines.

**Table 1:** The summary of iPS cells established from HLEC.

Condition	MOI	Number of HLEC seeded on MEF (×10^4^)	iPS-like colonies	Established iPS clones	Efficiencies (%)
A	4	31.8	5	0	0
B	20	28.5	29	3 (L1B41, L1B34)	0.0011
C	60	4.8	26	1 (L1C51)	0.0021
D	4	2.0	13	1	0.0050
E	20	10.0	6	0	0
F	60	30.0	2	0	0
	**Total**	107.1	81	5	0.0005

The multiplicity of lentiviral infection (MOI; 4–60) conditions and cell numbers of cultivated HLEC (2.0–31.8×10^4^ cells) were combined to establish iPS cells (conditions A–F). iPS cell colonies could be obtained only in conditions B–D. The efficiency of establishing iPS cells was approximately 0.0005%.

### Human iPS cell culture

Adult HDF-derived human iPS cell line 253G1 and 201B7 were obtained from RIKEN Bio Resource Center (Tsukuba, Ibaraki, Japan). 253G1, 201B7, and adult HLEC-derived human iPS cells (L1B41, L1C51, and L1B34) were maintained in culture using an MMC-treated mouse embryonic fibroblast (MEF) feeder layer in ES culture medium containing DMEM/F12 (Invitrogen, Carlsbad, CA) supplemented with 20% knockout serum replacement (KSR, Invitrogen), 0.1 mM 2-mercaptoethanol (2-ME, Invitrogen), 0.1 mM non-essential amino acid (NEAA, Invitrogen), and 4 ng/mL bFGF (Wako, Osaka, Japan) [20].

### Immunofluorescent staining

Human iPS cells were fixed in 4% paraformaldehyde (PFA) or cold methanol. Cells were washed with Tris-buffered saline (TBS, Takara Bio, Shiga, Japan) 3 times, and incubated with TBS containing 5% donkey serum and 0.3% Triton X-100, for 1 h to block nonspecific reactions. Cells were then incubated with following antibodies as appropriate: anti-Nanog (AF1997, R&D Systems, Minneapolis, MN), anti-Oct3/4 (AF1759, R&D Systems), anti-Sox2 (clone245610, R&D Systems), anti-SSEA-4 (clone MC-813-70, R&D Systems), anti-K14 (PRB-155P, Covance, Berkeley, CA), anti-Pax6 (AD2.35, Santacruz Biotechnology, Santa Cruz, CA), anti-p63 (4A4, Santacruz Biotechnology), K12 (N-16, Santacruz Biotechnology), K3 (AE5, PROGEN Heidelberg, Germany), or anti-α-Crystallin (SPA-224, StressGen Biotechnologies, Ann Arbor, MI) at 4°C overnight. Cells were washed twice with TBS and incubated with a 1∶200 dilution of their respective Alexa-Fluor® 350-, 488-, or 568-conjugated secondary antibodies (Molecular Probes, Eugene, OR) for 2 h at room temperature. For single or double immunostainings, the stained cells were counterstained with Hoechst 33342 (Molecular Probes) as a final step and observed by fluorescent microscopy (Axiovert200M; Carl Zeiss Jena Gmbh, Jena Germany). For all immunofluorescent experiments, slides treated with isotype-matched nonspecific IgG antibodies were used as isotype controls. The number of K12 and Pax6 double-positive corneal epithelial colonies per well (12-well plate) was counted.

### Microarray analysis

Total RNA was obtained from iPS cells using the RNeasy total RNA kit (Qiagen, Valencia, CA). Target preparation and microarray processing procedures were performed as described in the Affymetrix GeneChip Expression Analysis Manual (Affymetrix, Santa Clara, CA, USA). Data were analyzed by GeneSpring GX (Silicon Genetics, Redwood City, CA).

### Teratoma assay

For teratoma formation, 1×10^6^ cells of each human iPS cell clone (L1B41, L1C51, and L1B34) were injected into the testis of pentobarbital-anesthetized SCID mice (CLEA JAPAN, Tokyo, Japan). Teratomas were enucleated 4–8 weeks post-injection, fixed overnight in 10% formalin, and paraffin-embedded. The tissues were processed into 5-µm thick sections and then stained with hematoxylin and eosin (H&E staining).

### Corneal epithelial differentiation from human iPS cells

iPS cells were differentiated by the stromal cell-derived inducing activity (SDIA) method [21]. In brief, iPS cells were harvested by a dissociation solution containing 0.25% trypsin (Invitrogen) and 0.1% Collagenase type IV (Invitrogen), and then seeded on a gelatin-coated dish to eliminate MEF feeder cells for 1 h at 37°C. Non-attached cells (mainly iPS cell clumps) were harvested and re-seeded on MMC-treated PA6 feeder layer at a density of 35 clumps per 3.8 mm^2^ in GMEM (Invitrogen) media supplemented with 10% KSR, 1 mM sodium pyruvate, 0.1 mM NEAA, and 0.1 mM 2-ME (differentiation medium). Differentiation culture continued for 12–16 weeks.

### Real-time RT-PCR

Total RNA was obtained from differentiated iPS cells at each culture period using the RNeasy total RNA kit. Reverse transcription was performed using the SuperScript First-Strand Synthesis System for RT-PCR (Invitrogen) according to the manufacturer's suggested protocol, and cDNA was used as a template for PCR. Quantitative real-time RT-PCR was performed using the ABI Prism 7500 Fast Sequence Detection System (Applied Biosystems, Foster City, CA) according to the manufacturer's suggested protocol. Primer pairs and TaqMan® MGB probes labeled with 6-carboxyfluorescein (FAM) at the 5′-end and non-fluorescent quencher at the 3′-end were designed with Assay-by-Design™ (Applied Biosystems, GAPDH: Hs99999905_m1, Pax6: Hs00240871_m1, K12: Hs00165015_m1, K3: Hs00365080_m1, delta-N p63: Hs00978339_m1, and K14: Hs00559328_m1). The thermocycling program was performed as follows: an initial cycle at 95°C for 20 s, followed by 45 cycles of 95°C for 3 s and 60°C for 30 s. All assays were run in duplicate of 3 or more individual samples.

### DNA methylation assay

Genomic DNA (gDNA) was extracted from L1B41, 253G1, and 201B7 iPS cells, as well as from normal adult HDF (Takara Bio) and HLEC by FastPure DNA kit (Takara Bio Kyoto Japan). The methylation assay was carried out as previously described [22]. Each gDNA was bisulfite converted and enzymatically digested after amplification. The fragmented gDNA was applied to the HumanMethylation450 BeadChip (Illumina, San Diego, CA). The methylation level of each sample was calculated by the GenomeStudio® Methylation module (Illumina) as AVG-β corresponding to the ratio of the signal from the methylated allele to the sum of signal of the unmethylated allele and signal of the methylated allele. An AVG-β value of 0 corresponds to no methylation, and a value of 1.0 corresponds to 100% methylation at the specific CpG sites. Differentiation score (diffscore) represents expression differences between samples. Samples with a diffscore of greater than ±33 correspond to *p*-value<0.001.

### Statistical Analysis

Data are expressed as mean ± S.E. Statistical analysis was performed using the Mann-Whitney rank-sum test or Student's *t*-test. All statistics were calculated using SigmaPlot 11.0 (Systat Software, Inc., San Jose, CA).

## Results

### Transfection of Yamanaka 4 factors can reprogram HLECs

**Figure 1:**
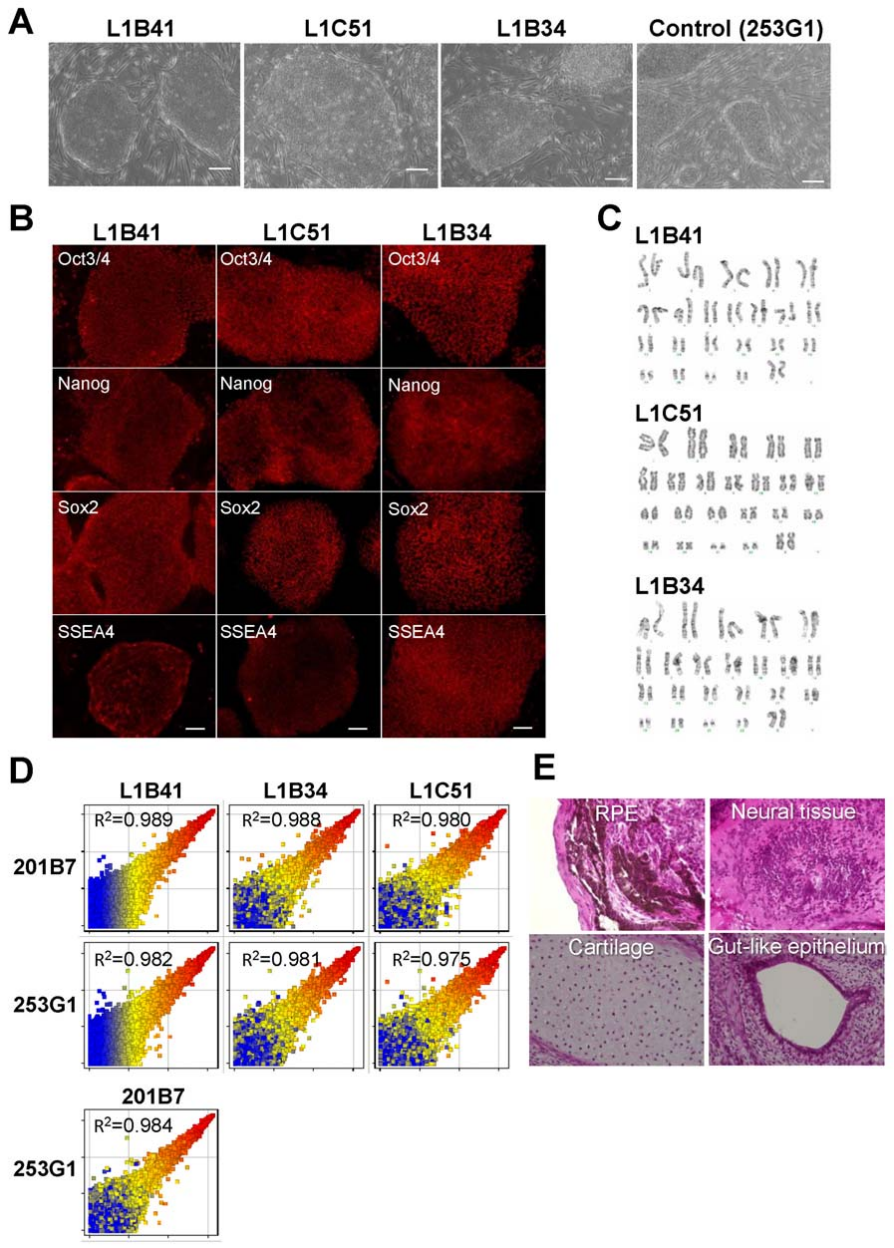
iPS cells were established from human corneal limbal epithelial cells (HLECs). (A) Three iPS-like cell colonies (L1B41, L1C51, and L1B34) were cloned after reprogramming of HLEC using Yamanaka 4 factors. (B) Immunofluorescent analysis showed that all 3 isolated iPS cell lines expressed the pluripotent stem-cell markers Nanog, Oct3/4, Sox2, and SSEA4. (C) Karyotype analysis showed no obvious aberration in the 3 iPS cell clones derived from HLEC. (D) Global expression analysis among iPS cells by microarray showed that the 3 HLEC-derived iPS cells exhibited similar expression to HDF-derived iPS cells. (E) HLEC-derived L1B41 was able to form a teratoma that contained the tissue originating from the 3 germ layers in the testis of SCID mice. Scale bar: (A) 200 µm, (B) 100 µm.

Since HLECs share the same cell lineage as corneal epithelial cells, we tested whether iPS cells derived from them would have a propensity to develop into corneal epithelial cells. To do this, a line of iPS cells was first established by transfecting HLECs with a lentiviral vector containing the 4 transcription factors Oct3/4, Sox2, c-Myc, and Klf4 (Yamanaka 4 factors) typically used to reprogram and induce pluripotency from already-differentiated somatic cells. We selected the iPS-like colonies and cultured them in iPS cell culture conditions. After 3–7 cell passages, several iPS cell lines were isolated (Table 1); among these, 3 HLEC-derived iPS cell clones (L1B41, L1B34, and L1C51) stably maintained their iPS-cell-like morphology throughout the cell passages, and were therefore selected for further testing (Fig. 1A). To examine whether the Yamanaka 4 factors induced expression of pluripotent stem cell markers in the selected HLEC-derived iPS cells, we analyzed Oct3/4, Nanog, SSEA-4, and Sox2 expression by immunostaining and found that all the 3 iPS cell lines expressed each of these pluripotent markers (Fig. 1B). The 3 iPS cell clones exhibited normal karyotype after several passages (Fig. 1C). Global expression analysis by microarray indicated that similar expression patterns were observed between HLEC- and HDF-derived iPS cells (Fig. 1D). Functional pluripotency was demonstrated in these HLEC-derived iPS cells, as the cultured L1B41 cells exhibited teratoma formation containing derivatives from all 3 germ layers, such as neural, gut, RPE, and cartilage tissues, 4–8 weeks after injection into mouse testis (Fig. 1E). Taken together, these data indicate that HLECs were successfully reprogrammed to iPS cells.

### Corneal epithelial cells can be differentiated from HLEC- and HDF-derived iPS cells

**Figure 2:**
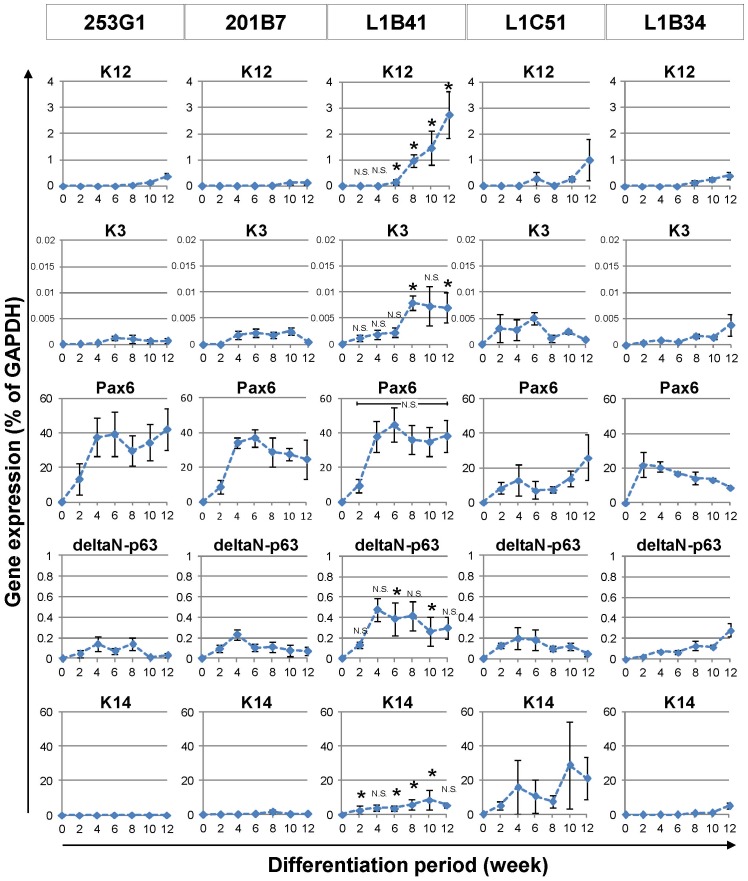
Gene expression analysis for corneal epithelium-related markers in iPS cells at various time points during differentiation by the SDIA method. While L1B41 began expressing significant levels of K12 after 6 weeks, other iPS cells began exhibiting K12 expression at approximately 10 weeks or later, and K12 expression in L1B41 was significantly higher than 253G1 at 6–12 weeks. Significant K3 expression levels were detected in L1B41 cells after 8 weeks in SDIA culture. Pax6 and delta-N p63 began to be expressed at 2 weeks and kept a similar expression pattern for several weeks in all iPS cells. K14 was highly expressed in both L1B41 and L1C51. The graph shows the mean ± S.E. of 3–7 independent samples. *p<0.05 (L1B41 vs. 253G1, Mann-Whitney rank-sum test), N.S. = not significant.

We next determined whether both the HLEC-derived iPS cells (L1B41, L1C51, and L1B34) and the commercially obtained HDF-derived iPS cells (253G1 and 201B7) could differentiate into corneal epithelial cells by the SDIA cell-differentiation method. Real-time RT-PCR analysis during each differentiation period showed that only the L1B41 cells began expressing significant levels of K12, a cytokeratin protein specific for corneal epithelium, after 6 weeks (Fig. 2). Similarly, K3, a corneal epithelial cell-specific keratin that pairs with K12, was significantly expressed after 8 weeks in L1B41 cells, even though the expression level appeared to be lower than K12. Although 253G1 and the other iPS cells also exhibited K12 expression after 10 weeks of culture, expression levels were significantly lower than L1B41. The expression pattern of Pax6, a marker of ocular and neural tissues, was similar among iPS cells. Additionally, L1B41 showed relatively higher levels of K14 and delta-N p63, markers of stratified epithelial cells, than 253G1 or 201B7. As K12 and K3 are the most specific corneal epithelial differentiation markers and not expressed in any other tissue besides corneal epithelium, the successful induction of K12 and K3 expression suggests that the SDIA method with long-term culture is sufficient to differentiate iPS cells into corneal epithelial cells.

**Figure 3:**
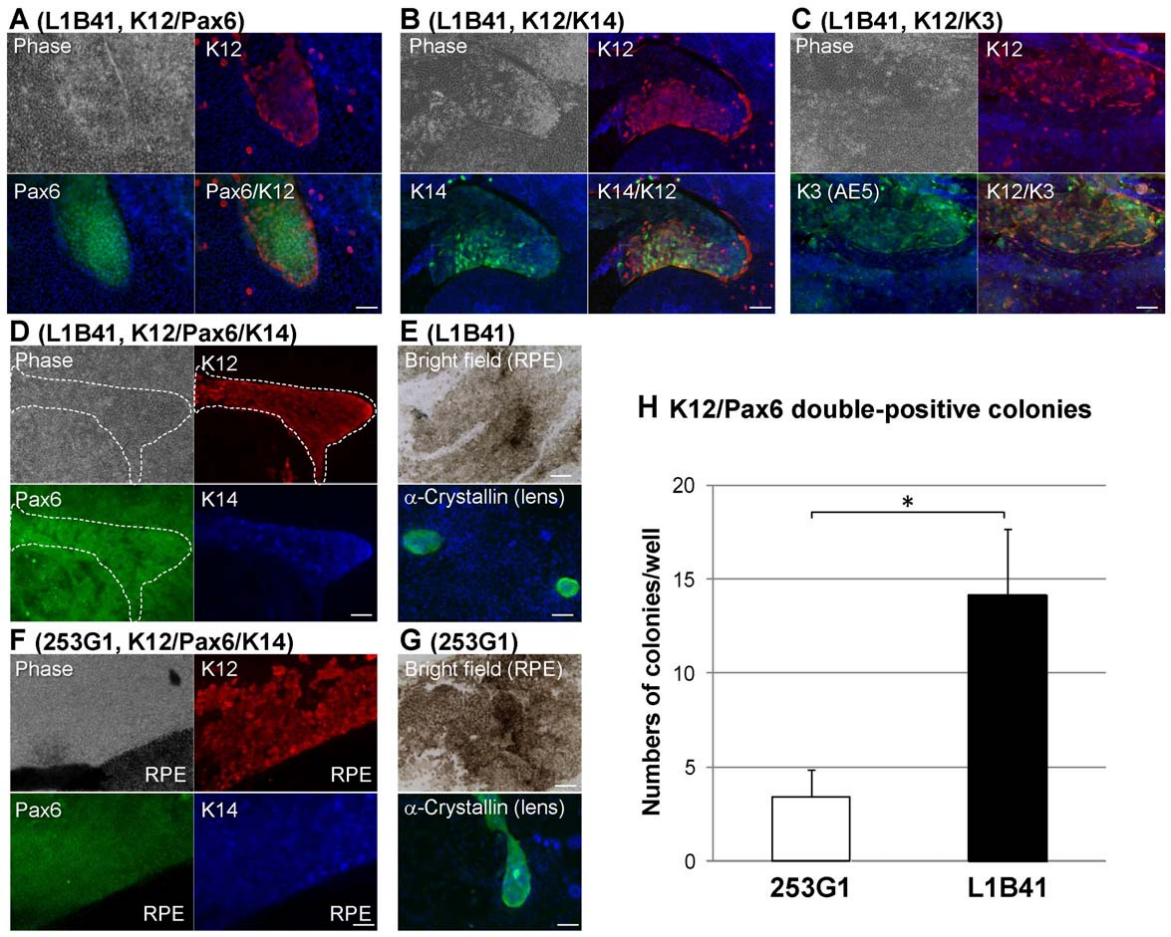
Immunofluorescence of corneal epithelial cell colonies induced from iPS cells. To examine corneal epithelial differentiation, double or triple immunostaining was performed. (A) Pax6 (green) and K12 (red) co-expressing colonies were observed in L1B41 cells differentiated for 12 weeks by the SDIA method. (B, C) Similarly, K12-expressing (red) colonies also co-expressed K14 and K3 (green) in L1B41 cells. (D) Furthermore, triple immunostaining showed that all 3 corneal epithelial differentiation markers co-localized in L1B41 (K12: red, Pax6: green, K14: blue). (F) Even in 253G1 iPS cells, corneal epithelial cell colonies were detected by immunostaining after approximately 15 weeks or later during the differentiation period (K12: red, Pax6: green, K14: blue). (E, G) The SDIA method was also able to induce RPE and lens epithelium (α-Crystallin^+^ cells) in both iPS cell lines. The number of corneal epithelial colonies (Pax6 and K12 double-positive) in L1B41 and 253G1 induced by SDIA method for 14 weeks were counted. (H) Corneal epithelial colonies in L1B41 were significantly higher than in 253G1. The graph showed the mean ± S.E. of 5 or 7 independent samples, respectively. Scale bar: 100 µm, *p<0.05 (*t*-test).

To further examine whether iPS cells had differentiated into corneal epithelial cells, we measured protein expression and localization by immunofluorescent staining. Double immunostaining for K12 and Pax6, K12 and K14, and K12 and K3 in the SDIA-differentiated cells derived from L1B41 at 12 weeks showed that K12-expressing cells appeared as a colony, and many of these cells co-expressed Pax6 in the nuclei (Fig. 3A), and similarly co-expressed K14 (Fig. 3B) and K3 (Fig. 3C) in the cytoplasm.

To verify K12, Pax6, and K14 co-localization, triple immunostaining was performed in the differentiated cells derived from both L1B41 and 253G1 iPS cells. The results showed that the K12^+^ colony also co-expressed Pax6 as well as K14 in both cell types (Fig. 3D, F). We also assayed for expression of markers from other ocular epithelia cell types and showed that the differentiated cells derived from both L1B41 and 253G1 also contained RPE (pigmented cell) and lens epithelium (α-Crystallin^+^ cell) in addition to the corneal epithelial cell markers (Fig. 3E–G). Statistical analysis revealed that the number of both Pax6- and K12-positive corneal epithelial colonies in L1B41 was significantly higher than in 253G1 (Fig. 3H). From these results, we conclude that long-term culture using the SDIA method can definitively induce corneal epithelium as well as RPE or lens epithelium from human iPS cells.

### BMP4 treatment suppressed corneal epithelial differentiation from iPS cells

**Figure 4:**
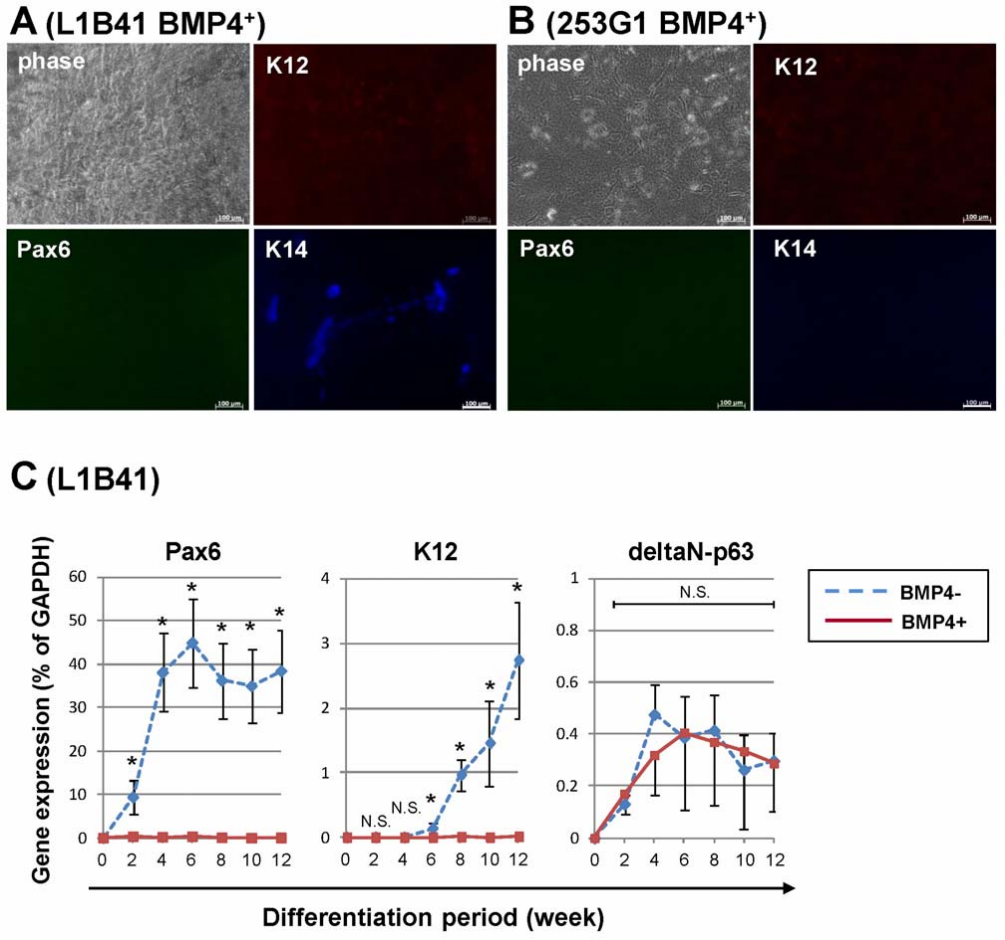
The effect of BMP4 treatment on iPS cells during corneal epithelial differentiation by the SDIA method. (A, B) 0.5 µM BMP4 was added during the first 2 weeks of differentiation by the SDIA method. Triple immunostaining showed that BMP4 treatment inhibited corneal epithelial induction from both iPS cell lines (K12: red, Pax6: green, K14: blue). (C) Real-time RT-PCR showed that Pax6 and K12 expression was completely suppressed by BMP4 treatment in L1B41 iPS cells. In contrast, BMP4 showed no significant effect on delta-N p63 expression. The graph showed the mean ± S.E. of 3–7 independent samples, respectively. *p<0.05 (BMP4^−^ vs. BMP4^+^, Mann-Whitney rank-sum test), N.S. = not significant. Scale bar: 100 µm.

BMP4 is known to promote surface ectodermal differentiation by suppressing neural differentiation. Since the corneal epithelium originates from surface ectoderm, we tested whether supplementing the SDIA method with BMP4 would improve differentiation of iPS cells to corneal epithelial cells. Unexpectedly, we observed almost no expression of Pax6 and K12 upon triple immunostaining for K12, Pax6, and K14 after BMP4 treatment in both SDIA-differentiated L1B41 and 253G1 iPS cells (Fig. 4A, B); some K14-expressing cells were observed in BMP4-treated L1B41 cells. Real-time RT-PCR confirmed that BMP4 treatment during differentiation completely suppressed Pax6 and K12 expressions in differentiated from L1B41 as compared to the SDIA method alone (Fig. 4C). There was no significant effect on delta-N p63 expression by BMP4 treatment. In contrast to what we predicted, these data suggest that the addition of BMP4 suppressed corneal epithelial cell differentiation from iPS cells.

### Genome-wide DNA methylation analyses

**Figure 5:**
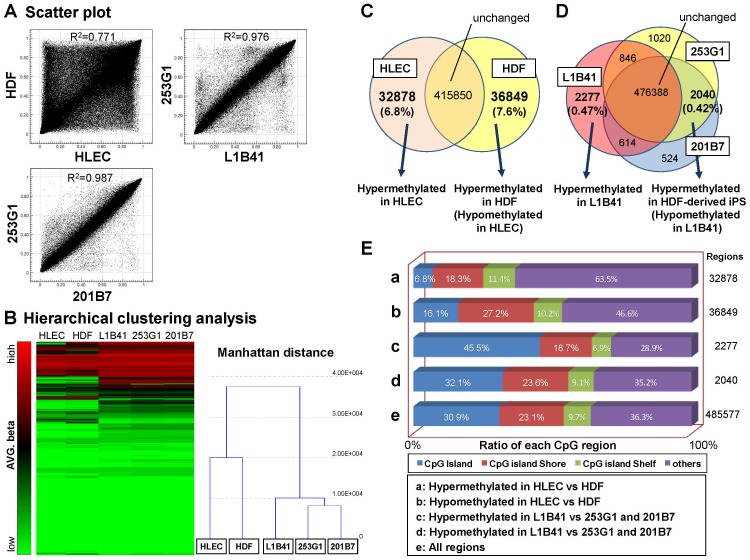
Genome wide DNA methylation analyses. (A) Scatter plots showed that a considerable difference in methylation status was observed between HLEC and HDF (A, R^2 = ^0.772). Between iPS cells (L1B41 vs. 253G1), the differences became smaller (R^2 = ^0.976), but were still larger than between 253G1 and 201B7 (R^2 = ^0.987). (B) The methylation status in HLEC, HDF and iPS cells was shown in the heatmap with green indicating unmethylated (low AVG-β) and red indicating fully methylated (high AVG-β). Hierarchical clustering analysis showed that L1B41 and 253G1 or 201B7 were differentially classified. (C) Comparing the methylation frequency between HLEC and HDF, 32878 hypermethylated regions (6.8%) and 36849 hypomethylated regions (7.6%) in HLEC were detected. (D) After reprogramming, 2277 hypermethylated regions (0.47%) and 2040 hypomethylated regions (0.42%) in L1B41 was detected by comparing between L1B41 and 253G1 or 201B7. (E) A large proportion of differentially methylated regions between HLEC and HDF were located in non-CpG islands (the ratio of CpG islands in hyper or hypomethylated regions in HLEC was 6.8% and 16.1%, respectively). Among iPS cells, a larger proportion of the differentially methylated regions were located in CpG islands (the ratio of CpG islands in hyper or hypomethylated regions in L1B41 was 45.5% and 32.1%, respectively).

**Figure 6:**
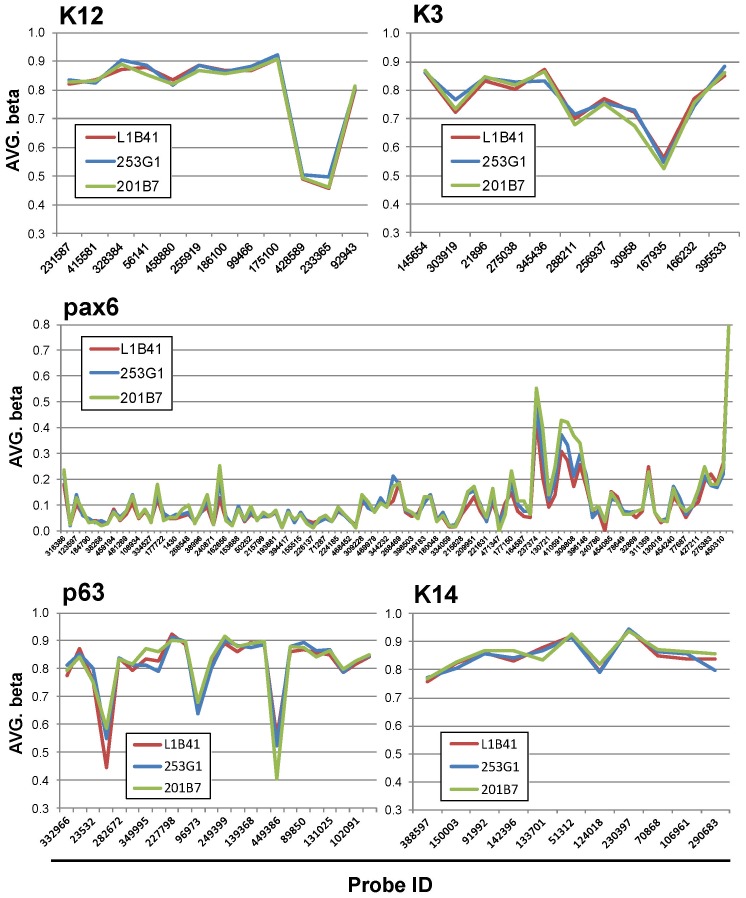
DNA methylation analysis of corneal epithelium-related genes. Methylation analysis of individual genes was performed. Methylation frequency in K12, K3, Pax6, p63, and K14 genes were not statistically different among L1B41, 253G1, and 201B7 (significance level; p<0.001 [ = diffscore >33 or <−33]).

It was previously suggested that the differentiation propensity of iPS cells was affected by epigenomic status [15], [16]. To examine whether the differential epigenomic status among iPS cells was related in the propensity of corneal epithelial differentiation, global DNA methylation status was examined by the HumanMethylation 450 BeadChip array, a newly designed high-density microarray used to quantify the methylation levels of over 450,000 CpG sites within the human genome. Scatter plot analysis showed that the difference in methylation status between L1B41 and 253G1 (R^2 = ^0.976) was much smaller than between their original cell types, HLEC and HDF (R^2 = ^0.771), even though the difference was larger than between 253G1 and 201B7 cells that were both derived from HDF cells (R^2 = ^0.987) (Fig. 5A). Hierarchical clustering analysis revealed that L1B41 was differentially classified from either 253G1 or 201B7 (Fig. 5B). The result of genome-wide methylation analysis revealed that 32878 hypermethylated regions (6.8%) and 36849 hypomethylated regions (7.6%) were present in the original HLEC cells as compared to the original HDF cells (Fig. 5C). Comparing the methylation status among the 3 iPS cells (L1B41, 253G1, and 201B7) revealed a drastic decrease in the differentially methylated regions upon reprogramming the original somatic cells (2277 hypermethylated regions [0.47%] and 2040 hypomethylated regions [0.42%] in L1B41 as compared to 253G1 and 201B7, respectively) (Fig. 5D). The ratio of CpG islands within the methylated regions between HLEC and HDF was relatively lower than in the other CpG-rich regions like the CpG-island shore or shelf (the hypermethylated regions were 6.8% and the hypomethylated regions were 16.1% in HLEC as compared to the 30.9% exhibited by all regions) (Fig. 5E). In contrast, the ratio of CpG islands became relatively higher between iPS cells (the hypermethylated regions were 45.5% and the hypomethylated regions were 32.1% in L1B41 as compared to the 30.9% exhibited by all regions) (Fig. 5E). Methylation analysis of individual genes revealed that no significant differences existed in the methylation status of the corneal epithelium-related markers K12, K3, Pax6, p63, and K14 genes among L1B41, 253G1, and 201B7 (Fig. 6).

## Discussion

Many studies show that cell lineages such as neurons, cardiac myocytes, or retinal cells can be differentiated from iPS or ES cells [23]–[25]. However, no established strategy for corneal epithelial cell has been described. In this study, we attempted to differentiate corneal epithelial cells from iPS cells derived from both HLEC and HDF, and were able to demonstrate the first known strategy to induce corneal epithelial cells from iPS cells. We also present data that L1B41 established from HLEC showed higher propensity for differentiation to corneal epithelium than other iPS cells. To accomplish this, we showed that introducing the Yamanaka 4 factors could reprogram HLECs. At least 3 iPS cell clones were successfully established using this method, and they exhibited typical pluripotent stem cell characteristics, such as Nanog expression, teratoma formation capability, and a normal karyotype. Microarray analysis showed that the 3 HLEC-derived iPS cell clones exhibited similar expression patterns to the HDF-derived 253G1 and 201B7 iPS clones with regard to complete suppression of differentiated corneal epithelial cell-related genes such as K12, K3, Pax6, delta-N p63, and K14. The efficiency of establishing iPS cells from HLECs was at least 0.0005% (Table 1), which was lower than iPS cells derived from other adult somatic tissues (0.0025–1.0%) [3], [10], [14]. To the best of our knowledge, this is the first report to establish human iPS cells from HLEC.

**Figure 7:**
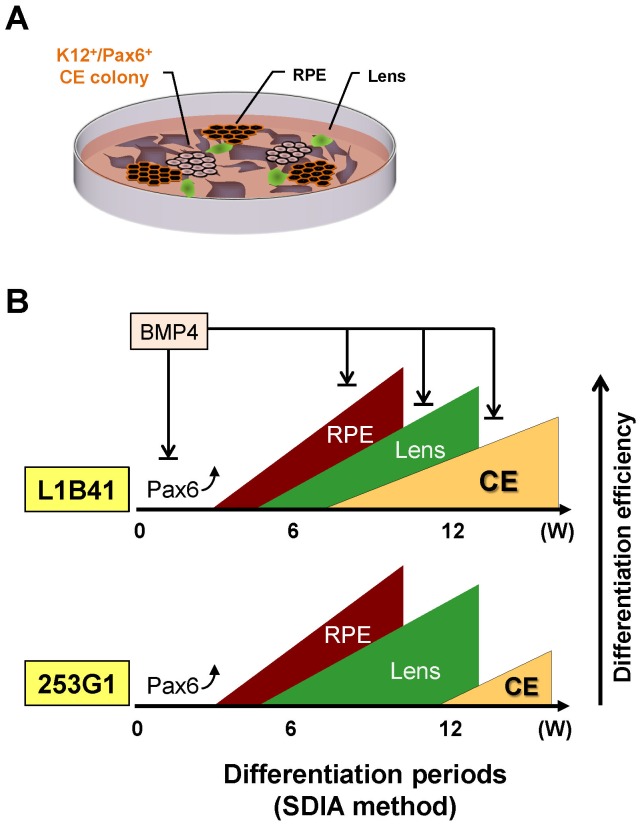
Schema for corneal epithelial induction from iPS cell by the SDIA method. (A) The basic SDIA method induced the serial differentiation of neuronal cell, RPE, lens and corneal epithelial cells (CE) from iPS cells in the same culture conditions. (B) Prolonged culture periods of the iPS cells in the SDIA culture method could also induce CE following RPE or lens epithelial cell induction. The efficiency and kinetics of appearance for corneal epithelial induction was different between 253G1 and L1B41. Early BMP4 treatment in SDIA method suppressed the differentiation of ocular cell lineages from iPS cells as well as Pax6 expression.

From the iPS cells we developed, we successfully differentiated corneal epithelial cells by the SDIA method as assessed by expression of the specific K12 and K3 cytokeratin protein markers that are exclusively expressed in differentiated corneal epithelium tissue [5], [26], [27]. Previous reports showed that the SDIA method could mainly induce neuro-ectodermal derivative cells such as dopaminergic neurons and RPEs [21], [25]. Our results showed that long-term SDIA differentiation culture could induce K12^+^/Pax6^+^ corneal epithelial cells after induction of differentiation of neural (data not shown), RPE, and lens cells (Fig. 3E, G). This is consistent with what is known to occur during ocular development, as corneal epithelial cells are thought to develop after RPE and lens cells and also are affected by these ocular tissues [28], [29]. Therefore, the data presented here along with previous data strongly suggest that the SDIA differentiation method *in vitro* mimics the processes of ocular cell development *in vivo*. As depicted in Figures 7A–B, RPE appeared at the beginning of SDIA-mediated differentiation after 4 weeks; subsequently, lens epithelium originated from surface ectoderm; finally, surface ectoderm-originated corneal epithelial cells were induced. Since corneal and lens epithelium originate during development from the same surface ectoderm *in vivo*, our data suggest that corneal epithelial cells and lens epithelial cells are also induced from the same origin cells during *in vitro* SDIA differentiation.

BMP signaling is known to promote surface ectodermal differentiation by suppressing neural differentiation [30], [31]. Even our own previous study also showed that BMP4 could promote the epithelial differentiation of murine ES or iPS cells [32]. We therefore used BMP4 as a supplement to the SDIA method in order to further promote corneal epithelial differentiation in the present study. Interestingly, our data showed that BMP4 completely suppressed corneal epithelial differentiation as well as RPE and lens epithelial cell differentiation. Likely, this suppression resulted from BMP4 inhibiting early Pax6 up-regulation that is essential for ocular cell development. Alternatively, BMP signaling has also reported to play an essential role in lens development [33], [34]. Although our data demonstrated that treatment with a dose of at least 0.5 µM BMP4 at an early time point during SDIA-mediated differentiation suppressed the development of ocular cells from iPS cells, the concentration and/or timing of BMP4 treatment may be important factors in ocular cell induction or development.

The most important discovery in this study is the significant difference we observed in the propensity for corneal epithelial cell differentiation among different iPS cells, as HLEC-derived L1B41 iPS cells exhibited higher K12 expression and larger corneal epithelial cell colony numbers than the HDF-derived 253G1 iPS cells. Previous studies suggested that iPS cells partially retain the property of the original cell type; in particular, they retain the epigenomic signature of their original cell even after reprogramming [15]–[17]. The global methylation analysis we performed detected definitive regions that were differentially methylated between the L1B41 and 253G1 iPS cells, even though the difference was much smaller than between the respective HLEC and HDF original cell lineages. In contrast, methylation analysis for the individual K12, K3, Pax6, p63, and K14 genes between L1B41 and 253G1 or 201B7 iPS cells showed that there was no significant difference in their methylation patterns. These results suggest that the iPS cell propensity for corneal epithelial cell differentiation was due to the difference in methylation status of genes up-stream of K12 rather than the K12 gene itself. Although we could not identify the specific regions or genes, we further analyzed the CpG status of the methylated regions and found that the tissue-specific differentially methylated regions between HLEC and HDF were mainly located in the non-CpG islands, as previously reported [35]–[37]. Interestingly, the ratio of deferentially methylated regions existing in CpG islands among iPS cells were higher than between HLEC and HDF, indicating that the methylation status in CpG islands was important in the inherent propensity for corneal epithelium differentiation.

Although we demonstrated that corneal epithelial cells were able to differentiate from iPS cells, our data indicated that not all of the HLEC-derived iPS cells preferentially differentiated into corneal epithelial cells by the SDIA method. This suggests the possibility that the differences observed in methylation status between iPS cells resulted from differences not only between the original cell types but also the between the cell clones or even those acquired during the reprogramming process. These possibilities suggest that choosing the most appropriate iPS cell clone for corneal epithelial differentiation may be possible by analyzing the methylation status of the specific region that contributes the most to the propensity for corneal epithelial differentiation. In fact, while the gene expression among L1B41, 253G1, and 201B7 was similar, the methylation status showed some differences between L1B41 and 253G1 or 201B7.

Alternatively, some recent reports indicated that, in addition to DNA methylation, chromatin modification (such as H3K4 or H3K27 methylation) might also be important for cell differentiation [38], [39]. Therefore, further investigation into analyzing histone modifications and finding the specific CpG regions that regulate K12 expression will be necessary to clarify the relationship between the propensity of iPS cells to differentiate into corneal epithelial cells and epigenomic status.

In summary, the present study is the first to show that corneal epithelial cells can be generated from human iPS cells. Our study not only contributes important new discoveries for the basic research fields of corneal epithelial development and regulation of K12 expression, but also introduces a strategy to develop corneal epithelial cells that has great potential in clinical regenerative medicine to treat damaged corneal epithelium.

## References

[1] Takahashi K, Yamanaka S (2006) Induction of pluripotent stem cells from mouse embryonic and adult fibroblast cultures by defined factors. Cell 126 (4) 663–676. 16904174 10.1016/j.cell.2006.07.024

[2] Takahashi K, Tanabe K, Ohnuki M, Narita M, Ichisaka T, et al (2007) Induction of pluripotent stem cells from adult human fibroblasts by defined factors. Cell 131 (5) 861–872. 18035408 10.1016/j.cell.2007.11.019

[3] Yu J, Vodyanik MA, Smuga-Otto K, Antosiewicz-Bourget J, Frane JL, et al (2007) Induced pluripotent stem cell lines derived from human somatic cells. Science 318 (5858) 1917–1920. 18029452 10.1126/science.1151526

[4] Kinoshita S, Adachi W, Sotozono C, Nishida K, Yokoi N, et al (2001) Characteristics of the human ocular surface epithelium. Prog Retin Eye Res 20 (5) 639–673 Review. 11470454 10.1016/s1350-9462(01)00007-6

[5] Schermer A, Galvin S, Sun TT (1986) Differentiation-related expression of a major 64K corneal keratin in vivo and in culture suggests limbal location of corneal epithelial stem cells. J Cell Biol 103: 49–62. 2424919 10.1083/jcb.103.1.49 PMC2113783

[6] Cotsarelis G, Cheng SZ, Dong G, Sun TT, Lavker RM (1989) Existence of slow-cycling limbal epithelial basal cells that can be preferentially stimulated to proliferate: implications on epithelial stem cells. Cell 57: 201–209. 2702690 10.1016/0092-8674(89)90958-6

[7] Tseng SC (1989) Concept and application of limbal stem cells. Eye 3: 141–157. 2695347 10.1038/eye.1989.22

[8] Nishida K (2003) Tissue engineering of the cornea. Cornea 22 (7 Suppl) S28–34. 14703705 10.1097/00003226-200310001-00005

[9] Samson CM, Nduaguba C, Baltatzis S, Foster CS (2002) Limbal stem cell transplantation in chronic inflammatory eye disease. Ophthalmology 109: 862–868. 11986089 10.1016/s0161-6420(02)00994-6

[10] Aasen T, Raya A, Barrero MJ, Garreta E, Consiglio A, et al (2008) Efficient and rapid generation of induced pluripotent stem cells from human keratinocytes. Nat Biotechnol 26 (11) 127612–127684. 10.1038/nbt.1503 18931654

[11] Kim JB, Zaehres H, Wu G, Gentile L, Ko K, et al (2008) Pluripotent stem cells induced from adult neural stem cells by reprogramming with two factors. Nature 454 (7204) 646–650. 18594515 10.1038/nature07061

[12] Loh YH, Agarwal S, Park IH, Urbach A, Huo H, et al (2009) Generation of induced pluripotent stem cells from human blood. Blood 113 (22) 5476–5479. 19299331 10.1182/blood-2009-02-204800 PMC2689048

[13] Conrad S, Renninger M, Hennenlotter J, Wiesner T, Just L, et al (2008) Generation of pluripotent stem cells from adult human testis. Nature 456 (7220) 344–349. 18849962 10.1038/nature07404

[14] Bar-Nur O, Russ HA, Efrat S, Benvenisty N (2011) Epigenetic memory and preferential lineage-specific differentiation in induced pluripotent stem cells derived from human pancreatic islet beta cells. Cell Stem Cell 9 (1) 17–23. 21726830 10.1016/j.stem.2011.06.007

[15] Kim K, Doi A, Wen B, Ng K, Zhao R, et al (2010) Epigenetic memory in induced pluripotent stem cells. Nature 467 (7313) 285–290. 20644535 10.1038/nature09342 PMC3150836

[16] Hu Q, Friedrich AM, Johnson LV, Clegg DO (2010) Memory in induced pluripotent stem cells: reprogrammed human retinal-pigmented epithelial cells show tendency for spontaneous redifferentiation. Stem Cells 28 (11) 1981–1991. 20882530 10.1002/stem.531

[17] Bock C, Kiskinis E, Verstappen G, Gu H, Boulting G, et al (2011) Reference Maps of human ES and iPS cell variation enable high-throughput characterization of pluripotent cell lines. Cell 144 (3) 439–452. 21295703 10.1016/j.cell.2010.12.032 PMC3063454

[18] Hayashi R, Yamato M, Takayanagi H, Oie Y, Kubota A, et al (2010) Validation system of tissue-engineered epithelial cell sheets for corneal regenerative medicine. Tissue Eng Part C Methods 16 (4) 553–560. 19722828 10.1089/ten.TEC.2009.0277

[19] Fujioka T, Shimizu N, Yoshino K, Miyoshi H, Nakamura Y (2010) Establishment of induced pluripotent stem cells from human neonatal tissues. Hum Cell 23 (3) 113–118. 20973836 10.1111/j.1749-0774.2010.00091.x

[20] Osakada F, Jin ZB, Hirami Y, Ikeda H, Danjyo T, et al (2009) In vitro differentiation of retinal cells from human pluripotent stem cells by small-molecule induction. J Cell Sci 122 (Pt 17) 3169–3179. 19671662 10.1242/jcs.050393

[21] Kawasaki H, Suemori H, Mizuseki K, Watanabe K, Urano F (2002) Generation of dopaminergic neurons and pigmented epithelia from primate ES cells by stromal cell-derived inducing activity. Proc Natl Acad Sci U S A 99 (3) 1580–1585. 11818560 10.1073/pnas.032662199 PMC122233

[22] Bibikova M, Barnes B, Tsan C, Ho V, Klotzle B, et al (2011) High density DNA methylation array with single CpG site resolution. Genomics 98 (4) 288–295. 21839163 10.1016/j.ygeno.2011.07.007

[23] Yokoo N, Baba S, Kaichi S, Niwa A, Mima T, et al (2009) The effects of cardioactive drugs on cardiomyocytes derived from human induced pluripotent stem cells. Biochem Biophys Res Commun 387 (3) 482–488. 19615974 10.1016/j.bbrc.2009.07.052

[24] Hirami Y, Osakada F, Takahashi K, Okita K, Yamanaka S, et al (2009) Generation of retinal cells from mouse and human induced pluripotent stem cells. Neurosci Lett 458 (3) 126–131. 19379795 10.1016/j.neulet.2009.04.035

[25] Kawasaki H, Mizuseki K, Nishikawa S, Kaneko S, Kuwana Y, et al (2000) Induction of midbrain dopaminergic neurons from ES cells by stromal cell-derived inducing activity. Neuron 28 (1) 31–40. 11086981 10.1016/s0896-6273(00)00083-0

[26] O'Guin WM, Galvin S, Schermer A, Sun TT (1987) Patterns of keratin expression define distinct pathways of epithelial development and differentiation. Curr Top Dev Biol 1987;22: 97–125 Review. 10.1016/s0070-2153(08)60100-3 2443318

[27] Hayashi R, Yamato M, Sugiyama H, Sumide T, Yang J, et al (2007) N-Cadherin is expressed by putative stem/progenitor cells and melanocytes in the human limbal epithelial stem cell niche. Stem Cells 25 (2) 289–296. 17008425 10.1634/stemcells.2006-0167

[28] Ashery-Padan R, Marquardt T, Zhou X, Gruss P (2000) Pax6 activity in the lens primordium is required for lens formation and for correct placement of a single retina in the eye. Genes Dev 14 (21) 2701–2711. 11069887 10.1101/gad.184000 PMC317031

[29] Collinson JM, Quinn JC, Buchanan MA, Kaufman MH, Wedden SE, et al (2001) Primary defects in the lens underlie complex anterior segment abnormalities of the Pax6 heterozygous eye. Proc Natl Acad Sci U S A 98 (17) 9688–9693. 11481423 10.1073/pnas.161144098 PMC55513

[30] Wilson PA, Hemmati-Brivanlou A (1995) Induction of epidermis and inhibition of neural fate by Bmp-4. Nature 376 (6538) 331–333. 7630398 10.1038/376331a0

[31] Mehler MF, Mabie PC, Zhang D, Kessler JA (1997) Bone morphogenetic proteins in the nervous system. Trends Neurosci 20 (7) 309–317 Review. 9223224 10.1016/s0166-2236(96)01046-6

[32] Sakurai M, Hayashi R, Kageyama T, Yamato M, Nishida K (2011) Induction of putative stratified epithelial progenitor cells in vitro from mouse-induced pluripotent stem cells. J Artif Organs 14 (1) 58–66. 21298309 10.1007/s10047-010-0547-3

[33] Furuta Y, Hogan BL (1998) BMP4 is essential for lens induction in the mouse embryo. Genes Dev 12 (23) 3764–3775. 9851982 10.1101/gad.12.23.3764 PMC317259

[34] Faber SC, Robinson ML, Makarenkova HP, Lang RA (2002) Bmp signaling is required for development of primary lens fiber cells. Development 129 (15) 3727–3737. 12117821 10.1242/dev.129.15.3727

[35] Doi A, Park IH, Wen B, Murakami P, Aryee MJ, et al (2009) Differential methylation of tissue- and cancer-specific CpG island shores distinguishes human induced pluripotent stem cells, embryonic stem cells and fibroblasts. Nat Genet 41 (12) 1350–1353. 19881528 10.1038/ng.471 PMC2958040

[36] Nagae G, Isagawa T, Shiraki N, Fujita T, Yamamoto S, et al (2011) Tissue-specific demethylation in CpG-poor promoters during cellular differentiation. Hum Mol Genet 20 (14) 2710–2721. 21505077 10.1093/hmg/ddr170

[37] Liang P, Song F, Ghosh S, Morien E, Qin M, et al (2011) Genome-wide survey reveals dynamic widespread tissue-specific changes in DNA methylation during development. BMC Genomics 12 (1) 231. 21569359 10.1186/1471-2164-12-231 PMC3118215

[38] Hawkins RD, Hon GC, Lee LK, Ngo Q, Lister R, et al (2010) Distinct epigenomic landscapes of pluripotent and lineage-committed human cells. Cell Stem Cell 6 (5) 479–491. 20452322 10.1016/j.stem.2010.03.018 PMC2867844

[39] Hirabayashi Y, Gotoh Y (2010) Epigenetic control of neural precursor cell fate during development. Nat Rev Neurosci 11 (6) 377–388 Review. 20485363 10.1038/nrn2810

